# Differential effects of lenalidomide during plasma cell differentiation

**DOI:** 10.18632/oncotarget.8581

**Published:** 2016-04-04

**Authors:** Michel Jourdan, Maïlys Cren, Peter Schafer, Nicolas Robert, Christophe Duperray, Laure Vincent, Patrice Ceballos, Guillaume Cartron, Jean-François Rossi, Jérôme Moreaux, Rajesh Chopra, Bernard Klein

**Affiliations:** ^1^ INSERM, U1040, Montpellier, France; ^2^ CNRS UPR1142, Institute of Human Genetics, Montpellier, France; ^3^ Translational Development Department, Celgene Corporation, Summit, NJ, USA; ^4^ CHU Montpellier, Laboratory for Monitoring Innovative Therapies, Department of Biological Hematology, Montpellier, France; ^5^ Cytometry IRB, Montpellier Rio Imaging, Montpellier, France; ^6^ CHU Montpellier, Department of Clinical Hematology, Montpellier, France; ^7^ University Montpellier 1, UFR Medicine, Montpellier, France

**Keywords:** plasma cell, differentiation, IKZF1, IKZF3, lenalidomide

## Abstract

Thalidomide, lenalidomide and pomalidomide have greatly improved the outcome of patients with multiple myeloma. However, their effects on plasma cells, the healthy counterpart of myeloma cells, are unknown. Here, we investigated lenalidomide effects on normal human plasma cell generation using an *in vitro* model. Lenalidomide inhibited the generation of pre-plasmablasts and early plasma cells, while it moderately affected plasmablast production. It also reduced the expression level of Ikaros, Aiolos, and IRF4 transcription factors, in plasmablasts and early plasma cells. This suggests that their differential sensitivity to lenalidomide is not due to a difference in Ikaros or Aiolos degradation. Lenalidomide also inhibited long-lived plasma cell generation, but did not impair their long-term survival once generated. This last observation is in agreement with the finding that lenalidomide treatment for 3-18 months did not affect the bone marrow healthy plasma cell count in allografted patients with multiple myeloma. Our findings should prompt to investigate whether lenalidomide resistance in patients with multiple myeloma could be associated with the emergence of malignant plasmablasts or long-lived plasma cells that are less sensitive to lenalidomide.

## INTRODUCTION

Thalidomide and its immunomodulatory derivatives (IMiDs®) lenalidomide and pomalidomide have greatly improved the outcome of patients with multiple myeloma (MM), a malignant plasma cell (PC) disorder [1–3]. IMiDs directly kill multiple myeloma cells (MMCs) and also target the tumor environment by stimulating T cell function and inhibiting angiogenesis and tumor necrosis factor production by monocytes [4]. These activities are mediated through IMiD ability to promote binding of the Ikaros and Aiolos transcription factors to cereblon (CRBN), which forms an E3 ubiquitin ligase complex together with damaged DNA binding 1 (DDB1), cullin-4A (CUL4A) and regulator of cullins 1 (ROC1) [5, 6]. This results in the activation of CRBN E3 ligase activity, leading to Ikaros and Aiolos ubiquitination and proteasomal degradation [7–9]. As Aiolos and Ikaros repress interleukin-2 (IL-2) production by T cells [10], their proteasomal degradation induced by lenalidomide releases this inhibition and enhances T cell stimulation [9].

The killing of MMCs by lenalidomide is partially explained by the down-regulation of interferon regulatory factor 4 (IRF4), a transcription factor essential for MMC survival [11, 12]. Recent works have shown that lenalidomide-induced IRF4 inhibition in MMCs occurs downstream of Ikaros/Aiolos reduction and suggest that IRF4 is a transcriptional target of Ikaros and/or Aiolos [7, 8, 13].

IMiD effects on PCs, the healthy counterpart of MMCs, are unknown. Aiolos has a critical role in the generation of bone marrow (BM) PCs in mice [14], and IMiDs could target human PC generation as well. Indeed, Aiolos-deficient mice are unable to generate high affinity antibodies upon T cell-dependent antigen vaccination, whereas their ability to form germinal center and memory B cells (MBCs) is unaffected [14]. The generation of BM PCs requires the sequential activation, differentiation and trafficking of B cells, plasmablasts and PCs in different compartments (lymph nodes, lymph, blood and BM) [15]. Circulating PBs are short-lived cells that must find a niche in the BM or mucosal tissues to provide them with the factors required to survive and to fully differentiate into BM PCs (about 0.25% of human BM cells) [15].

We and others have shown that PC generation can be modeled using multi-step culture systems where various combinations of activation molecules and cytokines are successively used to reproduce the sequential cell differentiation occurring in the different organs/tissues *in vivo* [16–20]. In these culture models, MBCs differentiate into CD20^low/−^CD38^−^ pre-plasmablasts (prePBs), CD20^−^CD38^+^CD138^−^ PBs, CD20^−^CD38^+^CD138^+^ early PCs and long-lived PCs (LLPCs), which may survive and produce continuously high amounts of immunoglobulins (Igs) for months *in vitro* [21, 22]. The phenotype of *in vitro-*generated PBs and early PCs is similar to the phenotype of the few PBs detected in the peripheral blood (2 PBs/mm^3^) [19, 23]. Moreover, the molecular events occurring during differentiation of B cells into PCs are recapitulated in these *in vitro* differentiation models. In prePBs, which secrete Igs weakly, PC transcription factors (*BLIMP1* and *XBP1*, mainly unspliced *XBP1* mRNA) start to be expressed, while *BCL6*, *PAX5* and other B cell transcription factors are progressively down-regulated. This change is more pronounced in early PCs (high Ig secretion) in which expression of *PAX5* and *BCL6* is inhibited and the ratio of spliced to unspliced *XBP1* mRNA is increased [20].

Using this model, here we show that lenalidomide mainly targets the generation of highly proliferating prePBs, poorly proliferating early PCs and non-proliferating LLPCs. Conversely, lenalidomide does not affect much the generation of proliferating PBs and does not alter the long-term survival of LLPCs, once generated. Despite the different sensitivity of PBs and early PCs to lenalidomide, the expression of lkaros and Aiolos is comparably reduced in both cell types upon incubation with this drug.

## RESULTS

### Sequential generation of long-lived plasma cells

**Figure 1:**
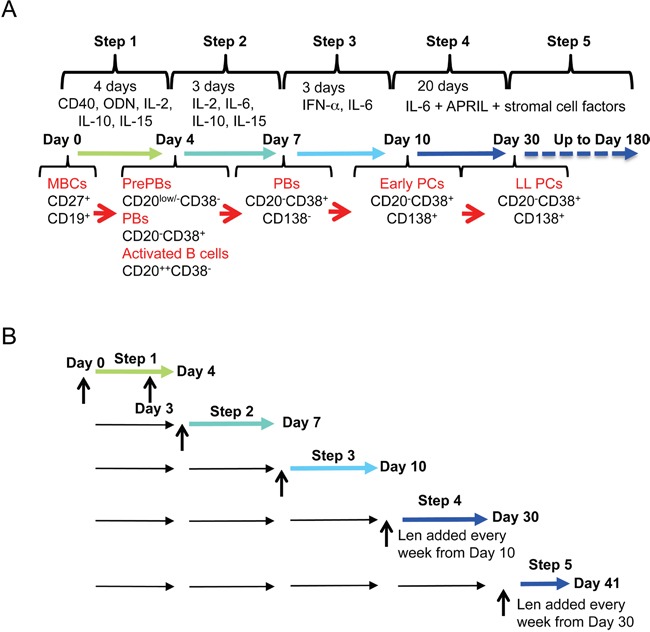
*In vitro* model to investigate lenalidomide effect during memory B cell differentiation into long-lived plasma cells **A.** Using a five-step culture system, human memory B cells are induced to differentiate into long-lived plasma cells from day 0 to day 30. They can then be maintained in culture up to day 180. The cytokines used and the phenotype of the obtained cell populations at each step are indicated. **B.** The effect of lenalidomide on the different populations (from memory B cells to plasma cells) is investigated at the end of each differentiation step. Vertical arrows indicate when lenalidomide is added.

To investigate the effect of lenalidomide on the generation of human LLPCs from MBCs, we used an *in vitro* model that mimics the various steps associated with this process in lymph nodes, blood and BM [19, 20, 22]. In step 1 (four days of culture with soluble CD40 ligand (CD40L), phosphorothioate CpG oligodeoxynucleotides (ODN), IL-2, IL-10 and IL-15), purified MBCs are activated and induced to differentiate into highly proliferating CD20^low/−^CD38^−^ prePBs that start to differentiate into CD20^−^CD38^+^ PBs [20]. In step 2, cells are cultured with IL-2, IL-10, IL-15 and IL-6, but without CD40L and ODN for three days (day 4 to 7) to promote differentiation into CD20^−^CD38^+^ PBs, which start to differentiate into poorly proliferating CD20^−^CD38^+^CD138^+^ early PCs. In step 3, cells are cultured in the presence of IL-6, IL-15 and interferon-alpha to complete PB maturation into CD20^−^CD38^+^CD138^+^ early PCs. In step 4 (addition of IL-6, APRIL and stromal cell-conditioned medium), early PCs finally differentiate into CD20^−^CD38^+^CD138^+^ non-cycling LLPCs and in step 5, newly generated LLPCs are allowed to survive and produce Igs continuously for months. Figure 1A–1B shows a schema of the culture model with the times of lenalidomide addition.

### Lenalidomide impairs the generation of proliferating pre-plasmablasts mainly by reducing the number of cell divisions

**Figure 2:**
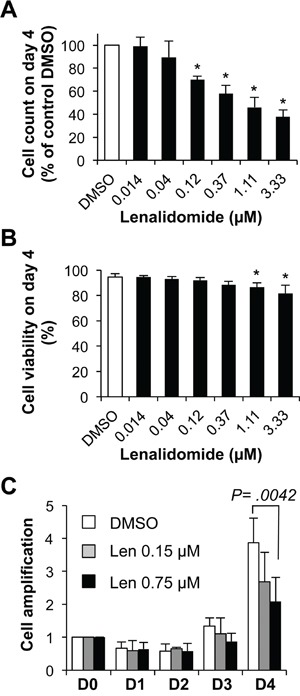
Effect of lenalidomide on cell growth in the first four days of *in vitro* memory B cell differentiation into plasma cells 1.5 × 10^5^ MBCs were activated with ODN and CD40L and cultured for four days with IL-2, IL-10, IL-15 and in the presence of increasing concentrations of lenalidomide or the largest DMSO concentration used to dilute lenalidomide (DMSO; control). **A.** Counts of cells recovered at the end of the four days of culture. **B.** Cell viability was assayed by trypan blue exclusion at day 4. **C.** Kinetics of cell amplification from D0 to D4, cells were cultured in the presence of control DMSO, 0.15 μM or 0.75 μM lenalidomide. Results are the mean value ± SD of four experiments. * *P* ≤ .05, compared with the control DMSO group using a paired t-test.

**Figure 3:**
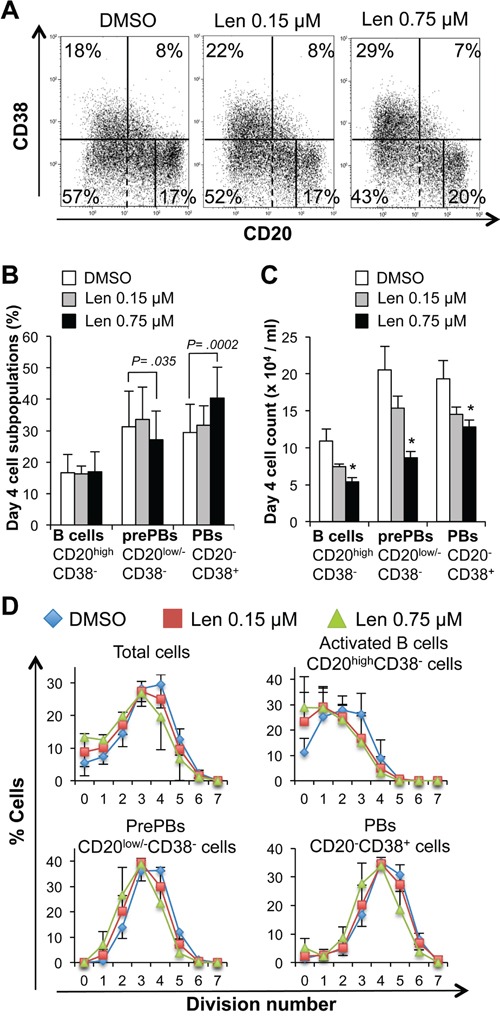
Lenalidomide reduces the generation of pre-plasmablasts in the first step of the B cell to plasma cell differentiation model MBCs were activated with ODN and CD40L and cultured for four days with IL-2, IL-10, IL-15 and in the presence of increasing concentrations of lenalidomide or the largest DMSO concentration used to dilute lenalidomide (DMSO; control). The percentage and count of activated B cells (CD20^high^CD38^−^), prePBs (CD20^low/−^CD38^−^) and PBs (CD20^−^CD38^+^) were determined by FACS analysis at day 4. **A.** Dot plot of CD20 and CD38 expression in day-4 cells (one representative experiment out of seven). The numbers in the panels (gates) are the percentages of gated day-4 cells. Dashed lines indicated the limit of CD20 positivity determined using an isotype-matched control antibody. **B** and **C.** Mean percentage and count ± SD (seven separate experiments) of activated B cells, prePBs and PBs at day 4 following incubation with 0.15 μM or 0.75 μM lenalidomide. **D.** Mean number (three separate experiments) of cell divisions to generate activated B cells, prePBs, or PBs at day 4. Cells were labeled with CFSE at the start of the culture and the decrease in CFSE staining due to cell division was evaluated at day 4. * *P* ≤ .05, compared with the control DMSO group using a paired t-test.

Addition of lenalidomide at the start of step 1 (day 0 to 4; differentiation of MBCs mainly into CD20^low/−^CD38^−^ prePBs and then CD20^−^CD38^+^ PBs) reduced the cell count (IC_50_ = 0.75 μM, a concentration in the range of those observed in patients treated with 25 mg lenalidomide daily) (Figure 2A) [24], but marginally reduced cell viability (Figure 2B). This effect was observed in the final day of step 1, when cells started cycling (Figure 2C). Moreover, 0.75 μM lenalidomide inhibited the generation of CD20^low/−^CD38^−^ prePBs by 58% compared to control cells (DMSO alone) (Figures 3A–3C). As cell viability was not affected, we investigated whether this inhibition was due to a reduction in the number of cycling and dividing cells. Indeed, the percentage of prePBs in S phase was decreased by 42% (45% of control cells *versus* 26% of cells incubated with 0.75 μM lenalidomide were in S phase) and the fraction of prePBs in G1 phase was increased by 34% (Supplementary Figure S1A). The mean number of cell divisions in prePBs was decreased by 17% (from 3.5 to 2.9 divisions) (Figure 3D and Table 1). Detailed flow cytometry data of a representative experiment are shown in Supplementary Figure S2.

**Table 1:** Lenalidomide reduces the number of cell division at day 4

Mean cell division number			
	DMSO	Lenalidomide 0.15 μM	Lenalidomide 0.75 μM
**Total cells**	3.2 ± 0.4	2.9 ± 0.5*	2.5 ± 0.7*
**Activated B cells** (CD20^high^CD38^−^)	2.0 ± 0.4	1.5 ± 0.5*	1.4 ± 0.5*
**PrePBs** (CD20^low/−^CD38^−^)	3.5 ± 0.2	3.2 ± 0.2*	2.9 ± 0.4*
**PBs** (CD20-CD38^+^)	4.1 ± 0.3	4.0 ± 0.4	3.5 ± 0.4

Mean number of cell divisions ± SD (three separate experiments) to generate activated B cells, prePBs, or PBs at day 4. Cells were labeled with CFSE at the start of the culture and the decrease in CFSE staining due to cell division was evaluated at day 4.

* *P* ≤ .05 compared with the control group (DMSO) using a paired t-test.

Concomitantly with the generation of CD20^low/−^CD38^−^ prePBs, MBC activation resulted also in the production of activated CD20^high^CD38^−^ B cells (17% of all day-4 cells) (Figures 3A–3C) as previously reported [20]. In the presence of 0.75 μM lenalidomide, the count of activated B cells was reduced by 51% compared to control cells (Figure 3C), as a result of the decrease in the fraction of cycling and dividing cells (Supplementary Figure S1B, Figure 3D and Table 1).

During step 1, some CD20^low/−^CD38^−^ prePBs further differentiated into CD20^−^CD38^+^ PBs (Figures 3A–3C). Since exposure to lenalidomide hindered the generation of prePBs and because PBs originate from this compartment,[20] PB count was also reduced in the treated cultures, compared with control (DMSO), at the end of step 1 (Figure 3C). However, the effect on PB count was about 2-fold lower than that on prePBs (34% *versus* 58% reduction, *P* ≤ .05). Similarly, the effect of 0.75 μM lenalidomide on the cell cycle was less strong in PBs than in prePBs: the PB fraction in S phase was reduced only by 17% compared with 42% in prePBs (*P* ≤ .05, Supplementary Figure 1A and 1C). Thus, lenalidomide mainly impairs prePB generation.

Lenalidomide did not significantly affect the frequency of IgM-, IgA- or IgG-producing activated B cells, prePBs and PBs (Supplementary Figure S3) or the expression of various membrane markers (CD19, CD24, CD30, CD45, CD126 and FCRL4). It only slightly increased CD31 expression in PBs (*P* = .009) (Supplementary Figure S4).

### Lenalidomide strongly inhibits the generation of CD138^+^ early plasma cells, but not of plasmablasts

**Figure 4:**
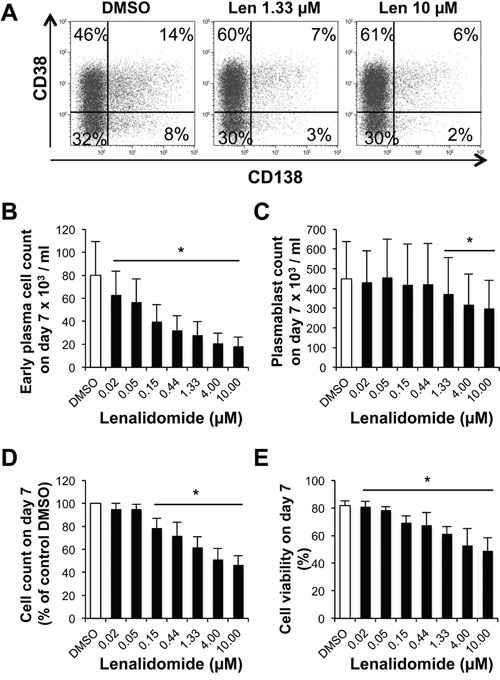
Lenalidomide preferentially targets the generation of CD138^+^ early plasma cells and poorly affects that of plasmablasts in the second step of *in vitro* B cell differentiation into plasma cells Cells harvested at the end of step 1 (day-4 cells: mainly prePBs and PBs) were cultured with IL-2, IL-6, IL-10 and IL-15 in the presence of graded concentrations of lenalidomide or the largest DMSO concentration used to dilute lenalidomide (DMSO) for three days. The percentage and counts of PBs (CD20^−^CD38^+^CD138^−^) and early PCs (CD20^−^CD38^+^CD138^+^) were determined by FACS analysis at day 7. **A.** Dot plot of CD38 and CD138 expression in day-7 cells (one representative experiment out of four). The numbers in the panels (gates) are the percentage of gated day-7 cells. **B** and **C.** Mean count ± SD of early PCs and PBs at day 7 in the presence of increasing lenalidomide concentrations from day 4 to day 7 (four separate experiments). **D** and **E.** Mean count and viability ± SD of all day-7 cells in the presence of increasing lenalidomide concentrations from day 4 to day 7 (four separate experiments). * *P* ≤ .05, compared with the DMSO control group using a paired t-test.

In step 2 (day 4 to 7), CD20^low/−^CD38^−^ prePBs differentiate into CD20^−^CD38^+^ PBs and then into CD20^−^CD38^+^CD138^+^ early PCs. Addition of lenalidomide at the beginning of this step led to a concentration-dependent reduction of early PC number at day 7 (50% reduction with 0.15 μM lenalidomide) (Figure 4A–4B). Conversely, PB number was considerably less affected. It was significantly reduced (by 17%) only upon incubation with 1.33 μM lenalidomide, a concentration which is about 10-fold higher than the early PC 50% inhibitory concentration (IC_50_) (*P* = .02, Figure 4C). Lenalidomide did not inhibit PB cell cycle at day 7 (Supplementary Figure S1D). The count and viability of all cells present in the cultures at day 7 were decreased, respectively, by 21% and 16% upon incubation with 0.15 μM lenalidomide and increasing lenalidomide concentrations had a stronger effect (Figures 4D–4E).

**Figure 5:**
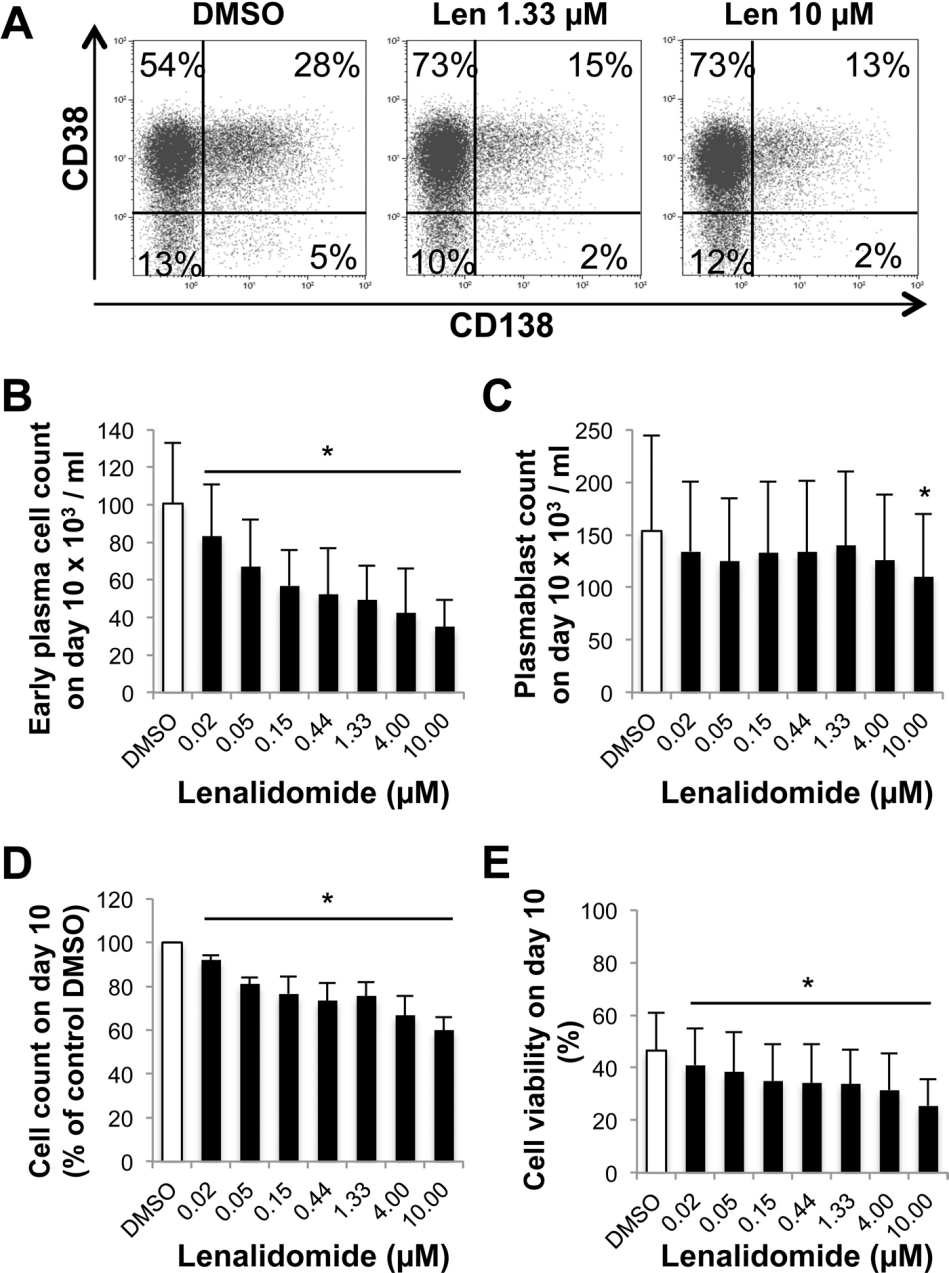
Lenalidomide preferentially targets the generation of CD138^+^ early plasma cells and poorly affects plasmablasts in the third step of the B cell to plasma cell differentiation model Cells harvested at the end of step 2 (day-7 cells, mainly PBs and early PCs) were cultured with IL-6, IL-15 and IFN- α for three days (step 3) in the presence of increasing concentrations of lenalidomide or the largest DMSO concentration used to dilute lenalidomide (DMSO; control). The percentage and counts of PBs (CD20^−^CD38^+^CD138^−^) and early PCs (CD20^−^CD38^+^CD138^+^) were determined by FACS analysis at day 10. **A.** Dot plot of CD38 and CD138 expression in day-10 cells (one representative experiment out of four). The numbers in the panels (gates) are the percentage of gated day-10 cells. **B** and **C.** Mean count ± SD of early PCs or PBs at day 10 in the presence of increasing lenalidomide concentrations from day 7 to day 10 (four separate experiments). **D** and **E.** Mean count and viability ± SD of all day-10 cells in the presence of increasing lenalidomide concentrations from day 7 to day 10 (four separate experiments). * *P* ≤ .05, compared with the DMSO control group using a paired t-test.

During step 3 (day 7 to day 10; maturation into CD20^−^CD38^+^CD138^+^ early PCs), incubation with lenalidomide reduced the generation of day-10 early PCs, with an IC_50_ of 0.76 μM (Figure 5A–5B). Conversely, it affected PB count only when used at a concentration higher than 4 μM (Figure 5C). The cell count and viability of all cells present in the culture at day 10 were decreased, respectively, by 27% and 28% with 0.76 μM lenalidomide and by 40% and 45% when lenalidomide concentration reached 10 μM (Figure 5D–5E). Thus, lenalidomide strongly inhibits the generation of CD138^+^ early PCs, but not of PBs in steps 2 and 3.

### Lenalidomide reduces the generation of long-lived plasma cells, but does not affect their survival once established

**Figure 6:**
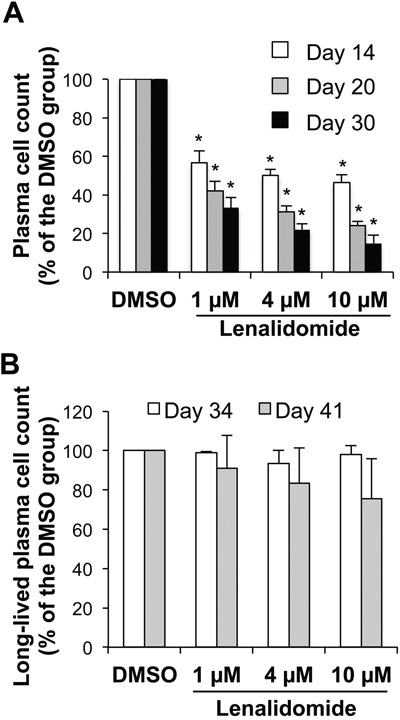
Lenalidomide targets the generation, but not the survival of long-lived plasma cells Early PCs generated at the end of step 3 (day-10 cells) were FACS-sorted based on CD138 expression and lack of CD20 expression (> 95% purity). **A.** Purified early PCs were cultured for 4, 10 or 20 days with IL-6, APRIL and stromal cell-conditioned medium (SC-CM) in the presence of increasing concentrations of lenalidomide or the largest DMSO concentration used to dilute lenalidomide (DMSO). Fresh lenalidomide, DMSO, culture medium, cytokines and SC-CM were refreshed every week by replacing half of the culture medium. Counts of metabolically active LLPCs were assayed at day 14, 20 and 30 using a Cell Titer Glo Assay. Data are expressed as the percentage (mean ± SD; n= three separate experiments) of the values obtained in controls (DMSO). **B.** 30-day LLPCs were incubated with increasing lenalidomide concentrations or the largest DMSO concentration used to dilute lenalidomide (DMSO) for 4 or 11 days. Fresh lenalidomide, control DMSO, culture medium, cytokines and SC-CM were refreshed every week, by replacing half of the culture medium. Counts of metabolically active LLPCs were assayed at day 34 and 41. Data are expressed as the percentage (mean ± SD; n= three separate experiments) of the values obtained in controls (DMSO). * *P* ≤ .05, compared with the control group (DMSO) using a paired t-test.

In step 4 (generation of CD20^−^CD38^+^CD138^+^ LLPCs), purified day-10 early PCs were cultured with IL-6, APRIL and stromal cell-conditioned medium (SC-CM) for 20 days. Lenalidomide was added at the beginning and then every week to ensure a continuous drug exposure according to the manufacturer's recommendations. In this step, cells poorly cycle and only 20% survive and differentiate into LLPCs [22]. Lenalidomide reduced the number of LLPCs at day 30 with an IC_50_ below 1 μM (Figure 6A). During step 5, newly generated LLPCs may survive for months, if fresh IL-6, APRIL and SC-CM are added every week [22]. Incubation of LLPCs harvested at day 30 with up to 10 μM lenalidomide for four (day 34) or 11 days (day 41) did not significantly affect the survival of established LLPCs (Figure 6B). Lenalidomide also did not modify their ability to produce IgG and IgA (Supplementary Figure S5A-S5B). These LLPCs did not cycle without or with lenalidomide (Supplementary Figure S1E).

### Ikaros and Aiolos are expressed in plasmablasts and early plasma cells and their expression is inhibited by lenalidomide

**Figure 7:**
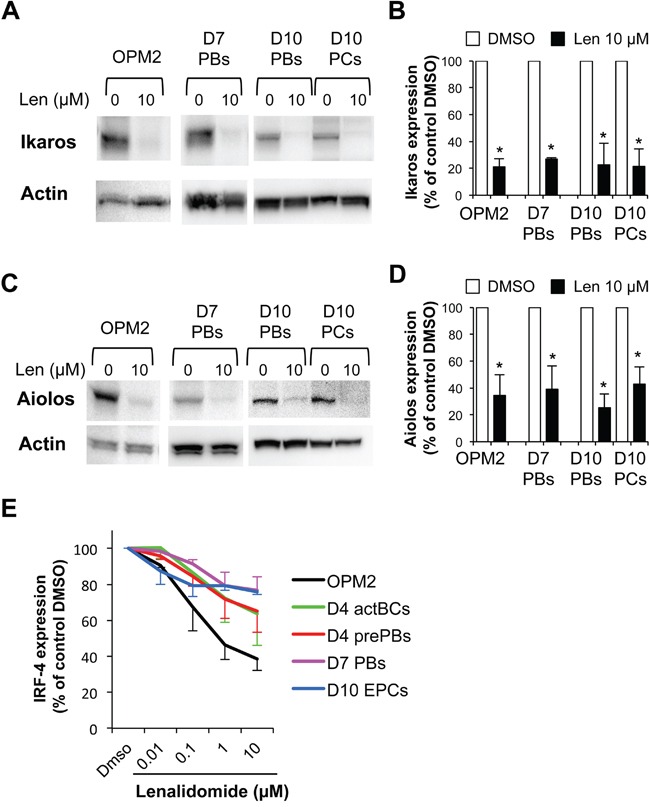
Ikaros, Aiolos and IRF4 are expressed in plasmablasts and early plasma cells and their expression is reduced by lenalidomide PBs generated at step 2 (day-7 cells), PBs and early PCs generated at step 3 (day-10 cells) were incubated with 10 μM lenalidomide or control DMSO for the last 24 hours of culture and were FACS-sorted based on CD38 expression and lack of CD20 and CD138 expression^19^ (PBs, ≥ 95% purity), and on CD138 and CD38 expression and lack of CD20 expression (early PCs ≥ 95% purity). OPM2 myeloma cells were incubated with 10 μM lenalidomide or control DMSO for 24 hours. Cell lysates were separated by SDS-PAGE and Ikaros and Aiolos expression assessed by immunoblotting. β actin expression was used as loading control. Representative western blot of Ikaros **A.** and Aiolos **C.** expression (three separate experiments). Data in **B** and **D.** are the mean Ikaros and Aiolos expression in the three experiments, quantified by densitometry analysis and normalized to actin levels. * P ≤ .05, compared with the control group (DMSO) using a paired t-test. **E.** Lenalidomide reduces IRF4 expression. Cells were cultured in the presence of increasing concentrations of lenalidomide or DMSO (control). Lenalidomide was added for three or four days at the beginning of each step, as indicated in Supplementary Figure S1B. The human myeloma cell line OPM2 was cultured for 3 days. At the end of each step, IRF4 expression was measured by flow cytometry analysis. Data are expressed as the percentage (mean ± SD; 3 separate experiments) of the IRF4 staining index values obtained in the control group (DMSO).

Ikaros and Aiolos expression levels in FACS-sorted day-7 PBs, day-10 PBs and day-10 early PCs were reduced by 70-80% and 50-65%, respectively, following incubation with lenalidomide (Figure 7A–7D). Similar results were obtained in OMP2 myeloma cells (positive control) treated with lenalidomide (Figure 7A–7D). This indicates that the differential sensitivity of PBs and early PCs to lenalidomide is not due to a difference in expression/targeting of Ikaros and Aiolos. The different sensitivity to lenalidomide could not be explained by differences in *CRBN* gene expression because it was similarly expressed in the various cell populations generated during plasma cell differentiation (Supplementary Figure S6A). CRBN protein expression levels, normalized to actin content, also were comparable in PBs and early PCs, despite the 10-fold higher sensitivity to lenalidomide of early PCs (Supplementary Figure S6B).

### Lenalidomide reduces IRF4 expression in activated B cell to plasma cell populations

Lenalidomide reduced in a dose-dependent manner IRF4 expression (measured by flow cytometry) in OPM2 myeloma cells (60% reduction with 10 μg/ml lenalidomide, Figure 7E and Supplementary Figure S7). It also significantly (P ≤ .05) reduced IRF4 expression in a dose-dependent manner in activated B cells, prePBs, PBs and early PCs (Figure 7E and Supplementary Figure S7). This effect was already significant with 0.1 μM lenalidomide and with 10 μM lenalidomide, IRF4 decrease varied from 23% in PBs to 36% in activated BCs. IRF4 reduction was comparable in PBs and early PCs with 1 μM and 10 μM lenalidomide (23% and 24% respectively). Interestingly, IRF4 downregulation was more pronounced in early PCs than in PBs with low doses of lenalidomide (0.01 μM and 0.1 μM) (Figure 7E).

### Treatment with lenalidomide does not affect the count of normal bone marrow plasma cells *in vivo*

**Figure 8:**
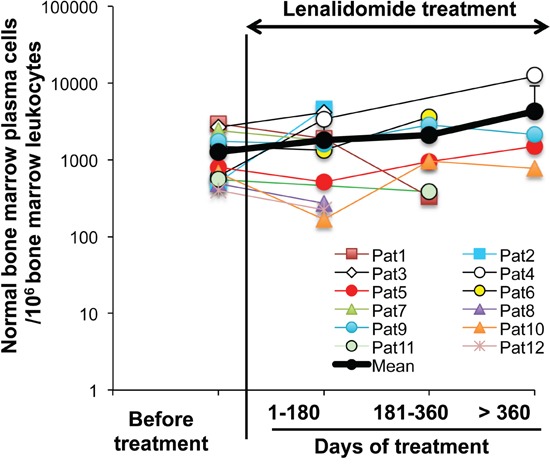
Lenalidomide treatment does not affect the counts of normal bone marrow plasma cells in allografted patients with multiple myeloma Twelve patients with MM who received an allogeneic hematopoietic stem cell transplant were treated with 25 mg lenalidomide daily for 3-18 months. Tumor and normal PCs were regularly monitored. All the patients had stable counts of tumor PCs during lenalidomide treatment. Normal PCs were identified as CD38^high^, CD19^+^, CD27^+^, CD56^−^, CD117^−^, CD200^−^ and cytoplasmic Kappa or Lambda Ig light chain^+^ cells using multicolor cytometry. Data are expressed as the normal PC counts/10^6^ leukocytes in the BM of each patient, harvested before and at different times during lenalidomide treatment. The black bold line represents the mean value of the patients' PC counts.

Twelve allografted patients with MM received 25 mg lenalidomide daily for 3 to 18 months following the detection of residual MMCs. Patients with increasing counts of malignant PCs during lenalidomide treatment were not considered in order to eliminate a possible competition between malignant and normal PCs for the same niche. BM MMCs and normal PCs were monitored regularly during the treatment. Although the number of BM PCs in each patient fluctuated over time, possibly due to BM sample variation, the mean count during the lenalidomide treatment did not differ from that before treatment (Figure 8).

## DISCUSSION

The main results of this study can be summarized in four points:

### Lenalidomide reduces the generation of highly proliferating pre-plasmablasts

We and others have shown that the differentiation of MBCs into prePBs and then PBs is a process requiring several cell divisions [17]. In agreement, here we found that prePBs are actively cycling (45% of prePBs in S phase). Lenalidomide impaired prePB formation by reducing the percentage of prePBs in S phase and increasing the fraction in G1 without affecting their viability. This is in line with the ability of lenalidomide to induce the cell cycle inhibitors p21^WAF1^ and p27^KIP1^ in tumor cell lines, particularly by promoting demethylation of the *p21^WAF1^* gene promoter [25].

### Lenalidomide poorly affects PB formation but dramatically inhibits the generation of early PCs

Lenalidomide reduced the early PC count with an estimated 50% inhibitory concentration of 0.76 μM, similar to the concentration that inhibits prePB formation. Conversely, lenalidomide began to affect PB generation only at 10-fold higher concentrations and did not impair their cell cycle. Early PCs are more differentiated than PBs. They are poorly cycling cells that express more cytoplasmic Igs, lack membrane Igs (PBs still express a low amount of membrane Igs) and strongly express CD27 and CD138 (syndecan-1) [19]. The primary targets of lenalidomide are the Ikaros and Aiolos transcription factors [7–9, 13]. However, the 10-fold difference in sensitivity of proliferating PBs and poorly proliferating early PCs to lenalidomide is not explained by differences in Ikaros and Aiolos expressions in these two cell types. Alternatively, lenalidomide could degrade more efficiently Ikaros or Aiolos in early PCs than in PBs. This was not the case when these cells were incubated with 10 μM lenalidomide (60% to 80% reduction in both cell types). However, we cannot exclude that these transcription factors might be degraded more efficiently in early PCs than in PBs when lower lenalidomide concentrations are used. We could not fully investigate this point because of the difficulty in obtaining enough cells to assess protein expression by western blotting at different time points and upon incubation with various lenalidomide concentrations. Moreover, the anti-Ikaros and-Aiolos antibodies, which are reported to be efficient for protein quantification by FACS, worked with myeloma cells in our hands, but not with the *in vitro* generated cell populations, probably due to the weak expression of these proteins in these cells. Nevertheless, this point should be further investigated when more efficient antibodies become available.

In myeloma cells, IRF4 is a major transcription factor for initiating plasma cell differentiation and Ikaros and Aiolos control IRF4 gene expression [7, 8, 13]. Lenalidomide also negatively modulates IRF4 expression as a consequence of the degradation of Ikaros and Aiolos [7, 8, 13]. In agreement, we found that lenalidomide degrades in a dose-dependent manner IRF4 in activated B cells, prePBs, PBs and early PCs. IRF4 degradation is much more pronounced in early PCs than in PBs treated with low doses of lenalidomide (i.e. 0,01 μM and 0,1 μM) but is comparable with higher doses (1 and 10 μM). At day-10 of culture, there is a significant decrease of cell viability affecting mainly early PCs [19]. IRF4 expression is critical for PC survival [11, 12], so we can hypothesize that early PCs surviving with high doses of lenalidomide are those selected according to high IRF4 expression. This could explain the absence of significant difference in IRF4 expression between early PC and PB at 10 μM lenalidomide concentration. Lenalidomide-induced IRF4 degradation in normal PBs and PCs is less pronounced than in OPM2 myeloma cells, possibly because IRF4 expression is deregulated in myeloma cells through continuous triggering by aberrant MYC expression [11, 12].

### Lenalidomide efficiently targets the generation of long-lived PCs from early PCs *in vitro*

Lenalidomide inhibits the generation of LLPCs starting from purified early PCs with an IC_50_ lower than 1 μM, a concentration in the range of those inhibiting prePB or early PC generation. The ability to generate LLPCs *in vitro* was recently documented by Cocco et al. and by our group [21, 22]. Our results fit well with the phenotype of Aiolos-deficient mice [14]. Indeed, they cannot generate PCs that produce Igs for a prolonged time [14]. As lenalidomide specifically targets Ikaros and Aiolos, these *in vivo* [14] and *in vitro* (our study) findings suggest that Aiolos and/or Ikaros are critical for LLPC generation from early PCs. In the current study, we could not determine Ikaros and Aiolos expression in LLPCs because of the too limited number of available LLPCs for western blot analysis. Indeed, only a few LLPCs can be generated due to the high cell loss occurring during the early PC and LLPC generation steps (80% of differentiating cells will die) [19, 22]. Moreover, although Ikaros and Aiolos immunodetection by FACS was previously reported in myeloma cell lines [13], we did not obtain specific labeling in PBs or early PCs and therefore could not use this approach in LLPCs (results not shown).

### Once LLPCs are generated, lenalidomide does not affect their long-term survival and Ig production

The *in vitro* generation of LLPCs from early PCs is a highly selective process, but once LLPCs are generated, they can efficiently survive for months *in vitro* [21, 22]. The current data indicate that Ikaros and Aiolos are not essential for LLPC survival in our *in vitro* differentiation system. Moreover, LLPC survival might not be affected by lenalidomide also *in vivo*. Indeed, in patients with MM who received a stem cell transplant, lenalidomide treatment for 3 to 18 months following detection of residual tumor cells did not affect the mean count of normal BM PCs, identified by flow cytometry. These data suggest that Ikaros and/or Aiolos are dispensable for the long-term survival of human LLPCs *in vitro* and *in vivo*. It should be interesting to investigate the changes in MBCs, prePBs, PBs and PCs in patients treated with lenalidomide.

In conclusion, this study shows that lenalidomide affects minimally the formation of human PBs, specifically targets the generation of prePBs from MBCs, of early PCs from PBs and of LLPCs from early PCs, and does not alter the survival of LLPCs once generated, in agreement with the lack of effect on normal BM PCs in patients treated with lenalidomide. As lenalidomide specifically targets Ikaros and Aiolos proteins, these data suggest a major role of these transcription factors in regulating the generation of early PCs and LLPCs in humans.

**Figure 9:**
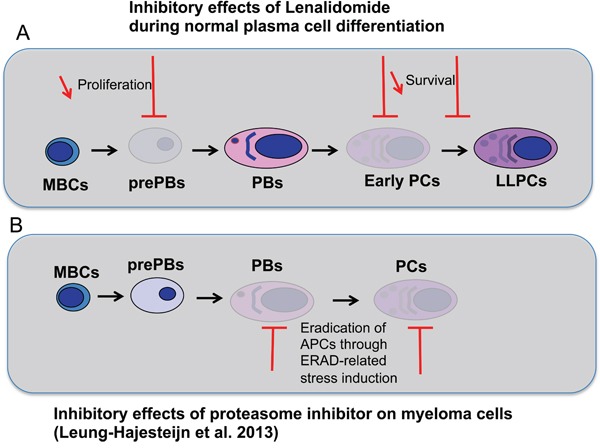
Effects of lenalidomide and proteasome inhibitor on the different stages of plasma cell differentiation **A.** During plasma cell differentiation, lenalidomide inhibits the generation of prePBs, early PCs and LLPCs but has a moderate inhibitory effect on PBs and LLPCs once generated. **B.** As shown by Leung-Hagesteijn et al. in multiple myeloma [28], antibody-producing cells (APCs), PBs and PCs, are sensitive to proteasome inhibitor because of their high level of Ig synthesis. Proteasome inhibitor treatment induces endoplasmic reticulum-associated protein degradation (ERAD)-related stress.

The specific targeting of plasma cell precursors by lenalidomide should prompt to investigate whether resistance of plasma cell tumors to lenalidomide is associated with the selection of plasmablast or mature plasma cell subclones. Multiple myeloma is a heterogeneous tumor the growth of which is supported by MM progenitors that differentiate into mature MMCs [26]. The phenotype of MM progenitors that generate tumors in immune-compromised mice is controversial, particularly their expression of CD138 [26, 27], the marker that discriminates early PCs (sensitive to lenalidomide) from PBs (poorly sensitive) in the current report. A recent study has shown that the proteasome inhibitor bortezomib kills preferentially mature and plasmablastic MMCs and that bortezomib resistance is associated with the emergence of pre-plasmablastic MMCs [28] (Figure 9B). The current data suggest that the MMC phenotype in patients resistant to lenalidomide should be investigated to determine whether resistance is associated with the emergence of poorly proliferating long-lived plasma cells or of CD38^+^CD138^−^ plasmablasts. In this case, the clinical benefit of lenalidomide and bortezomib association could be explained by the ability of this drug combination to kill pre-plasmablastic up to mature MMCs (Figure 9A–9B).

## MATERIALS AND METHODS

### Patients

This study was performed under approval of the Montpellier University Hospital Centre for Biological Resources (DC-2008-417). It included patients with multiple myeloma (MM) who received an allogeneic hematopoietic stem cell transplant after reduced intensity conditioning (fludarabine, intra-venous busulfan ± thymoglobulin) at the Department of Hematology, Montpellier University Hospital, France. They all signed a written informed consent according to the Helsinki Declaration and EBMT good clinical practice guidelines. Residual tumor disease was monitored using flow cytometry as indicated [29]. In the case of detection of ≥ 10^3^ MMCs/L, patients (n=12) received 25 mg lenalidomide daily for 3-18 months with repeated monitoring of MMCs and normal PCs.

### Reagents

Human recombinant interleukin (IL)-2 was purchased from R&D Systems (Minneapolis, MN, USA), interferon-alpha-2b (IFN- α, IntronA) from Merck Canada Inc. (Kirckland, Canada), IL-6, IL-10 and IL-15 from PeproTech (Rocky Hill, NJ, USA). Mouse monoclonal antibodies (mAbs) conjugated to allophycocyanin (APC), fluorescein isothiocyanate (FITC), peridinin chlorophyll protein-cyanin 5.5 (PerCP-Cy5.5), phycoerythrin (PE) or Pacific Blue^™^ (PB), and specific for human CD19 (clone HIB19), CD24 (clone ML5), CD27 (clone M-T271), CD30 (clone BerH8), CD31 (clone WM59), CD38 (clone HIT2), CD56 (cloneB159), CD117 (clone 104D2), CD138 (clone RF8B2), IgG (clone G18-145), IgM (clone G20-127), lambda Ig light chain (clone JDC-12), kappa Ig light chain (clone TB28-2) and Ki67 (clone B56) were purchased from BD Biosciences (Le Pont De Claix, France); for CD200 (clone OX104) from eBiosciences (San Diego, CA, USA); for CD20 (clone B9E9), CD45 (clone J33), CD126 (clone M91) and CD138 (clone B-A38) from Beckman Coulter (Fullerton, CA, USA); for IRF4 (clone IRF4.3E4) and FCRL4 (clone 580810) from BioLegend (San Diego, CA, USA); for IgA, IgM, and IgG (polyclonal goat Abs) from Southern Biotech (Birmingham, AL, USA).

### Cell samples

Peripheral blood cells from healthy volunteers were purchased from the French Blood Center (Toulouse, France) and CD19^+^CD27^+^ MBCs were purified (≥ 95% purity) as described [19].

### Cell cultures

MBCs were differentiated using a previously described five-step culture method [19, 20, 22]. All cultures were performed in Iscove modified Dulbecco medium (Invitrogen, Carlsbad, CA, USA) and 10% FCS. In step 1 (day 0 to day 4), 1.5 × 10^5^/ml purified peripheral blood MBCs were seeded in 6-well culture plates and were activated for 4 days by 10 μg/ml of phosphorothioate CpG oligodeoxynucleotides (ODN) 2006 (Sigma-Aldrich, St Louis, MO, USA), 50 ng/ml histidine tagged soluble CD40 ligand (CD40L) and 5 μg/ml of an anti-poly-histidine mAb (R&D Systems) in the presence of 20 U/ml IL-2, 50 ng/ml IL-10 and10 ng/ml IL-15. In step 2 (day 4 to day 7), PBs were generated by removing ODN and CD40L and changing the cytokine cocktail (20 U/ml IL-2, 50 ng/ml IL-6, 50 ng/ml IL-10 and 10 ng/ml IL-15). In step 3 (day 7 to day 10), PBs were differentiated into early PCs by adding 50 ng/ml IL-6, 10 ng/ml IL-15 and 500 U/ml IFN- α. In step 4 (day 10 to day 30), early PCs were FACS sorted based on CD138 expression and lack of CD20 expression (> 95% purity) and differentiated into LLPCs by adding 10 ng/ml IL-6, 100 ng/ml APRIL and 50% of stromal cell-conditioned medium (SC-CM). Fresh culture medium, growth factors and SC-CM were added once per week and cultures were maintained until day 30. In step 5 (day 30 to day 41), LLPCs were allowed to survive and produce continuously Igs in the presence of 10 ng/ml IL-6, 100 ng/ml APRIL and 35% of SC-CM. The SC-CM was obtained by culturing confluent monolayers of Resto-6 SC cells for 5 days. The culture supernatant was filtered with 0.2 μM filters and frozen. The Resto-6 cell line was obtained from lymph node-derived SCs, as previously described [30].

Various lenalidomide concentrations (Celgene Corporation, Summit, NJ, USA) were added at the start of each step and its effects evaluated by analyzing cell counts and phenotype at the end of each step. Supplementary Figure S1A-S1B shows a schema of the culture model with the times of lenalidomide addition.

### Cell viability and cell growth assays

Cell concentration and viability were assessed with the trypan blue dye exclusion test. The number of metabolically active cells was determined by intracellular ATP quantitation with the Cell Titer Glo Luminescent Assay (Promega Corporation, Madison, WI, USA).

### Cell cycle and immunophenotypic analysis

Cycling cells were identified by DAPI staining (Sigma-Aldrich) and cells in the S phase by incubation with bromodeoxyuridine (BrdU) for 1 hour followed by labeling with an anti-BrdU antibody (APC BrdU flow kit, BD Biosciences) according to the manufacturer's instructions. For immunophenotypic analysis, cells were stained with a combination of 4 to 7 mAbs conjugated to different fluorochromes, as indicated [22]. The Cytofix/Cytoperm kit (BD Biosciences) was used for intracellular staining of IgM, IgA, IgG, IRF-4 or Ki67 antigen [19]. Flow cytometry analysis was performed with a FACSAria cytometer using FACSDiva 6.1 (Becton Dickinson, San Jose, CA, USA) and with a Cyan ADP cytometer driven by the Summit software (Beckman Coulter). The Kaluza software (Beckman Coulter) was used for data analysis. The fluorescence intensity of the cell populations was quantified using the staining index (SI) formula: [mean fluorescence intensity (MFI) obtained for a given mAb minus MFI obtained with a control mAb]/[2 times the standard deviation of the MFI obtained with the same control mAb] [19].

### CFSE labeling

Cell division was assessed by CFSE labeling as previously described [31]. Briefly, purified MBCs were washed and re-suspended at a concentration of 10^6^ cells/ml in PBS/0.1% BSA with 10 μM CFSE (Molecular Probes, Eugene, OR, USA), incubated at 37°C for 10 minutes and extensively washed before culture. At day 4 of culture, cells were washed and labeled with anti-PB-CD20 and anti-PerCP-Cy5.5-CD38 for flow cytometry analysis. Cell divisions were quantified using the ModFit LT software (Verity Software House, Topsham, ME, USA).

### Western blot analysis

Cells were lysed in RIPA buffer (Cell Signaling Technology, Beverly, MA, USA) supplemented with 1 mM phenylmethylsulfonyl fluoride immediately before use. Lysates were separated by sodium dodecyl sulfate-polyacrylamide gel electrophoresis (10% gels) and transferred to nitrocellulose membranes using an iBlot® Gel Transfer Device (InVitrogen). Non-specific membrane sites were blocked by incubation at room temperature in 140 mM NaCl, 3 mM KCl, 25 mM Tris-HCl (pH 7.4), 0.1% Tween 20 (tris-buffered saline Tween-20), 5% non-fat milk for 2 h, and then immunoblotted with rabbit polyclonal antibodies against Ikaros (Santa Cruz Biotechnology, Dallas, TX, USA), Aiolos (Cell Signaling Technology) or Cereblon (Sigma-Aldrich). As a control for protein loading, a mouse monoclonal anti-β-actin antibody (Sigma-Aldrich) was used. The primary antibodies were visualized with peroxidase-conjugated goat anti-rabbit (Sigma-Aldrich) or goat anti-mouse (Jackson ImmunoResearch, West grove, PA, USA) antibodies and an enhanced chemiluminescence detection system. Western blots were quantified by densitometry using the NIH ImageJ software (National Institutes of Health, Bethesda, MD, USA) and protein levels were normalized according to those of β-actin.

### Analysis of Ig secretion

**ELISA**. Flow cytometry-sorted PCs (10^6^ cells/ml) were cultured for the indicated time with increasing lenalidomide concentrations or the largest DMSO concentration used to dilute lenalidomide (DMSO) and culture supernatants harvested. IgA and IgG concentrations were assessed by ELISA using human IgA and IgG ELISA kits from Bethyl Laboratories (Montgomery, TX, USA), according to the manufacturer's recommendations.

### Detection of malignant and normal PCs in BM of patients with MM

Malignant and normal PCs were counted using the multi-parameter flow cytometry technique reported previously [29]. Briefly, erythrocyte-lysed BM samples were labeled with anti-CD19, CD20, CD38 and CD45 monoclonal antibodies (mAbs) in association with anti-CD138, CD27, CD56, CD117, CD200 mAbs or isotype controls. Cells were then fixed and permeabilized with the Cytofix/Cytoperm kit (BD Biosciences, Le Pont De Claix, France), and labeled with anti-Ig Kappa and anti-Ig Lambda mAbs. Data were acquired with a Cyan flow cytometer, driven by the Summit 4.3 software (Beckman Coulter, Fullerton, CA, USA). B-lymphocytes were defined as [CD19^+^CD20^+^CD45^+^CD38^−/+^ and (Kappa^+^ or Lambda^+^)] cells and PCs as [CD38^high^ and (Kappa^+^ or Lambda^+^) and (not B lymphocytes)] cells. MMCs were identified based on the monoclonal expression of Kappa or Lambda light chains together with the aberrant expression of one or several myeloma markers: CD20 and/or CD56 and/or CD117 and/or CD200, lack of CD19, and lack/weak CD27 and/or CD45 expression. [32–35] Healthy PCs were identified based on the polyclonal expression of Kappa or Lambda light chains together with the expression of CD19, CD27. The acquisition of at least 100 events was required to define MMCs or healthy PCs as detectable [36]. Data were analyzed with the FlowJo 9.1 software (Tree star, Ashland, OR).

### Statistical analysis

Statistical comparisons were made with the non-parametric Mann-Whitney test, unpaired or paired Student's *t*-test using the SPSS software. *P*-values ≤ .05 were considered as significant.

## SUPPLEMENTARY FIGURES


