# Supplementary material for: Differential effects of lenalidomide during plasma cell differentiation
Source: Oncotarget. 2016 Apr 4;7(19):28096–111. doi: 10.18632/oncotarget.8581 (PMC5053712; doi:10.18632/oncotarget.8581)
Supplement: Supplementary file 1 [file oncotarget-07-28096-s001.pdf]

Differential effects of lenalidomide during plasma cell differentiation

SUPPLEMENTARY FIGURES

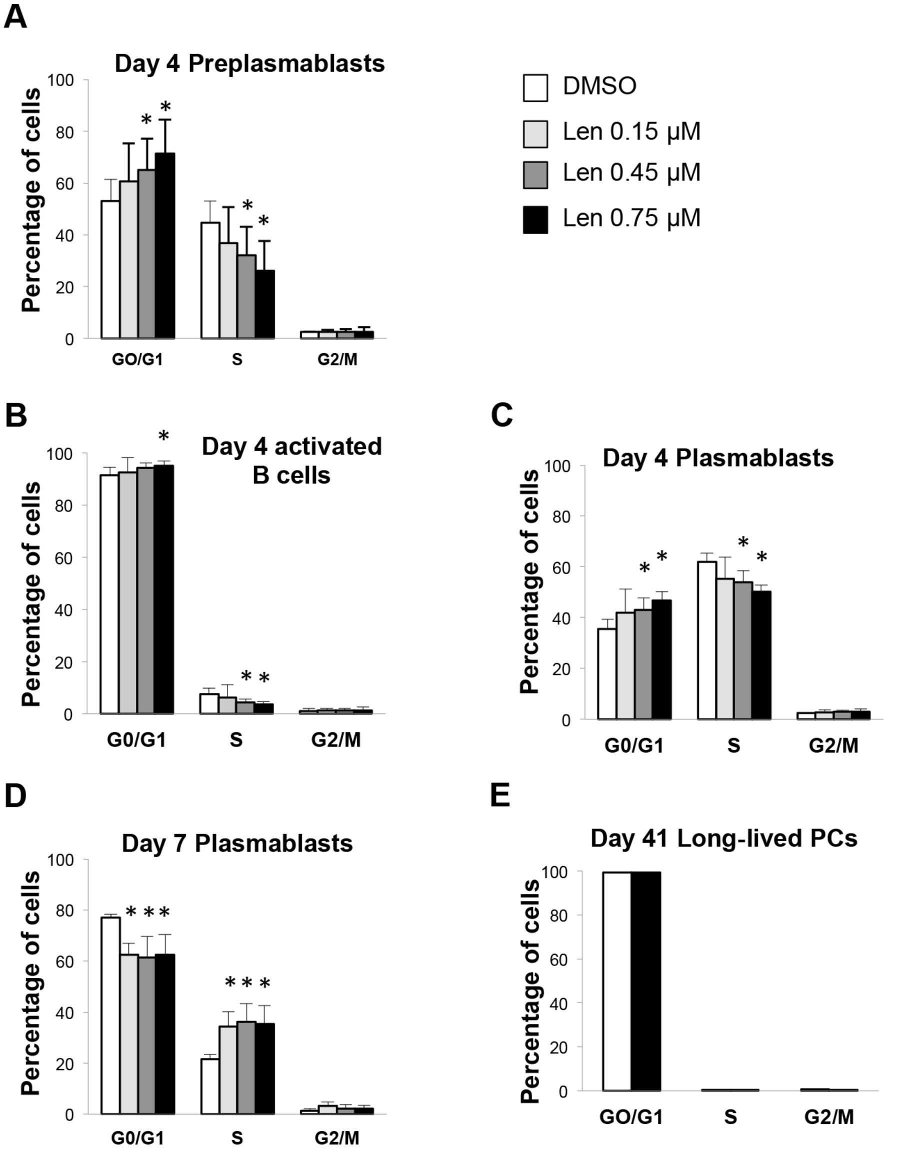

Supplementary Figure S1: Effect of lenalidomide on the cell cycle distribution of pre-plasmablasts, plasmablasts and long-lived plasma cells. MBCs were induced to differentiate in the 5-step culture system and cells were harvested at day 4, 7 and 41. Lenalidomide (0.15, 0.45 or 0.75 µM) or DMSO was added at the start of each step. The cell cycle distribution was assessed by DAPI staining and cells in S phase were quantified by bromodeoxyuridine (BrdU) incorporation and labeling with an anti-BrdU antibody. Dot plots show the percentage of cells in the G0/G1, S and G2/M phases in **A.** day-4 prePBs, **B.** day-4 activated B cells, **C.** day-4 PBs, **D.** day-7 PBs and **E.** day-41 LLPCs. Results are the mean percentage ± SD of cells in the various phases of the cell cycle in four separate experiments. * $P \leq .05$ compared with the control group (DMSO) using a paired t-test.

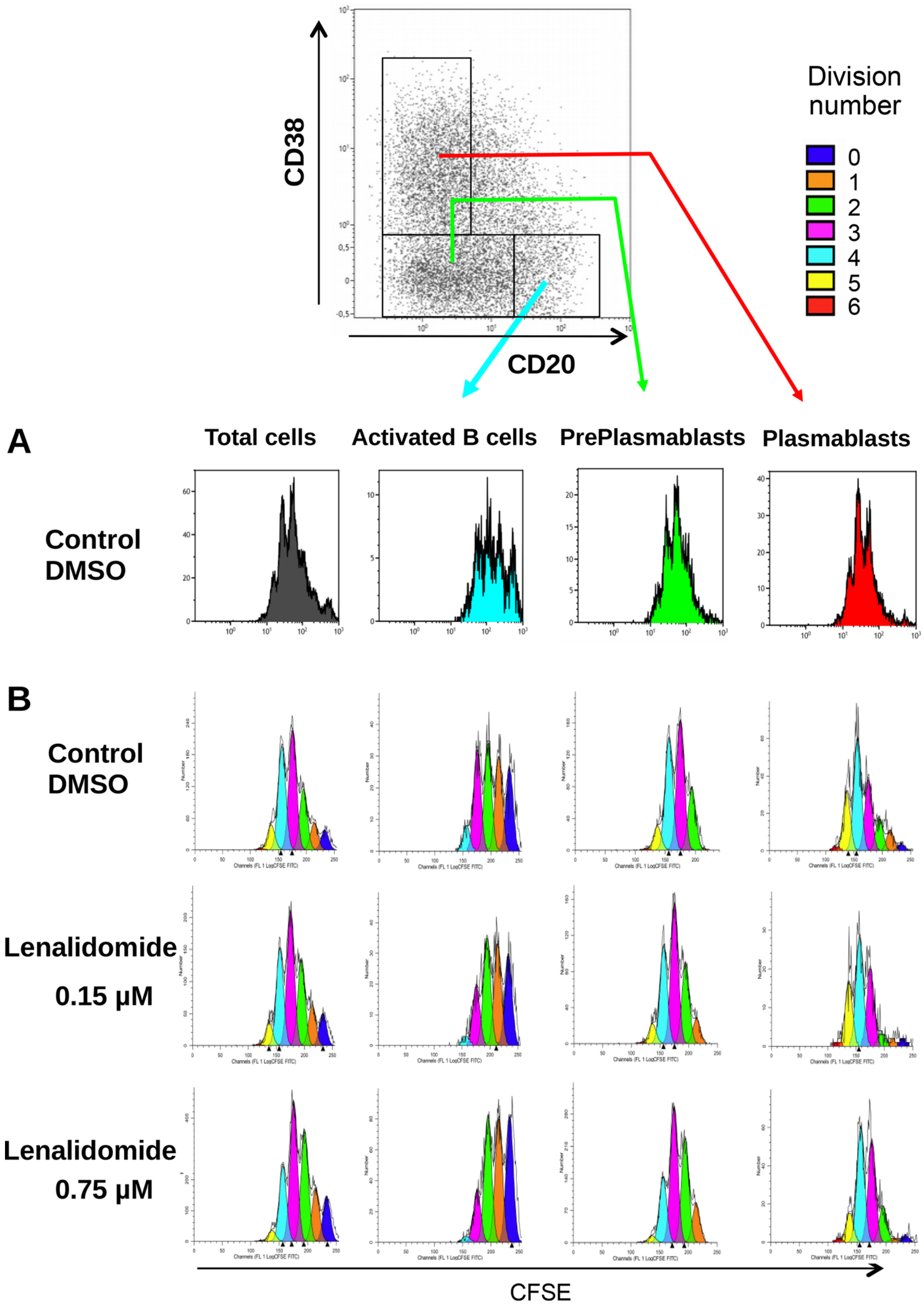

Supplementary Figure S2: The number of cell divisions in activated B cells, pre-plasmablasts, and plasmablasts at day 4 was assessed by CFSE labeling. A. Cells were labeled with CFSE at the start of the culture and at day 4, the decrease of CFSE staining due to cell divisions was measured in each subpopulation by gating. **B.** Histograms were processed with the ModFit Lt software to quantitate cell division numbers.

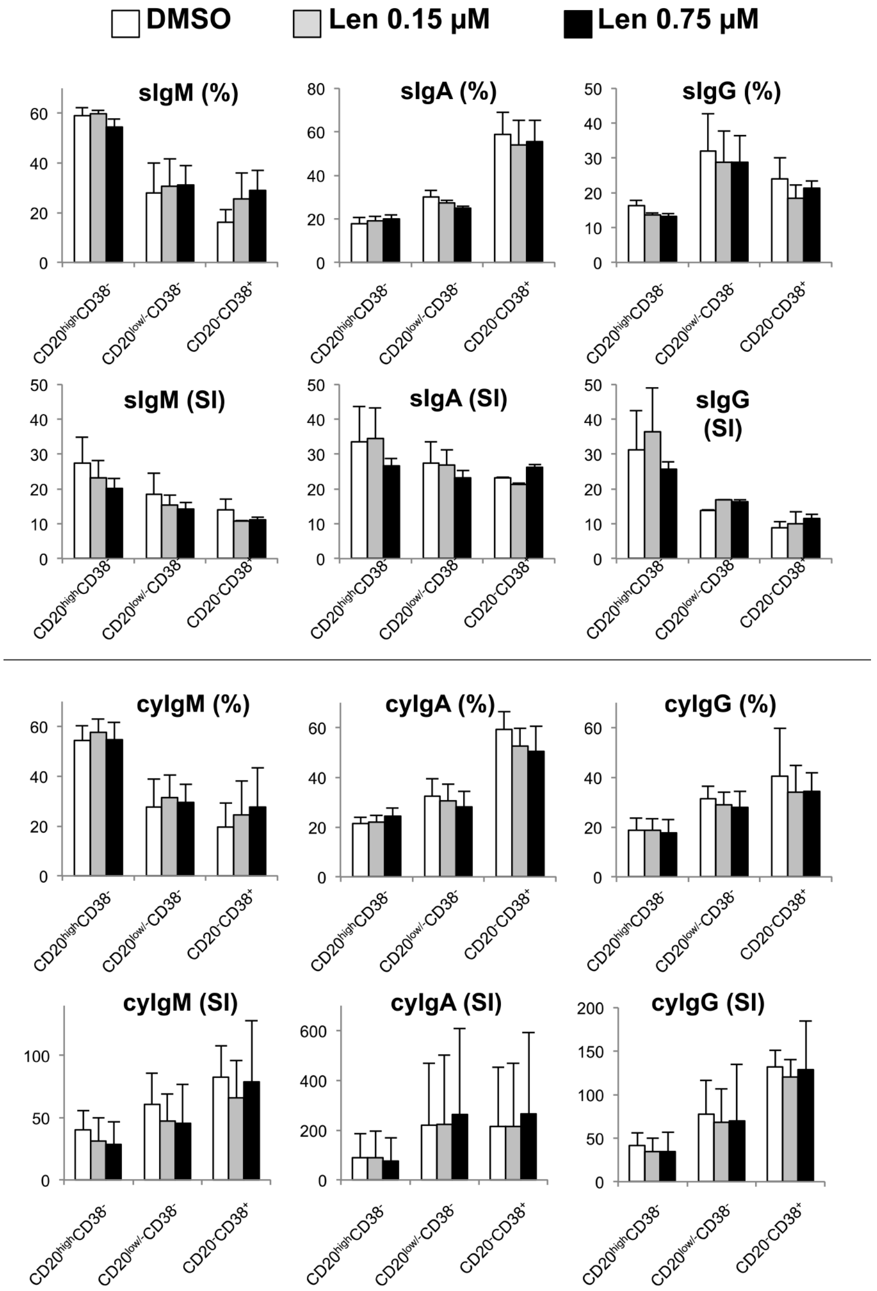

Supplementary Figure S3: Lenalidomide does not modify the surface and cytoplasmic expression of IgG, IgA and IgM in B cells, pre-plasmablasts and plasmablasts. MBCs were activated with ODN and CD40L and cultured with IL-2, IL-10, IL-15, in the presence of increasing concentrations of lenalidomide or the largest DMSO concentration used to dilute lenalidomide (DMSO; control). After four days, surface (s) and cytoplasmic (cy) IgG, IgA and IgM were determined in activated B cells ($CD20^{high}CD38^{-}$), prePBs ($CD20^{low/-}CD38^{-}$) and PBs ($CD20^{-}CD38^{+}$) by FACS analysis. Results are the mean percentage and staining index (SI) ± SD of three separate experiments.

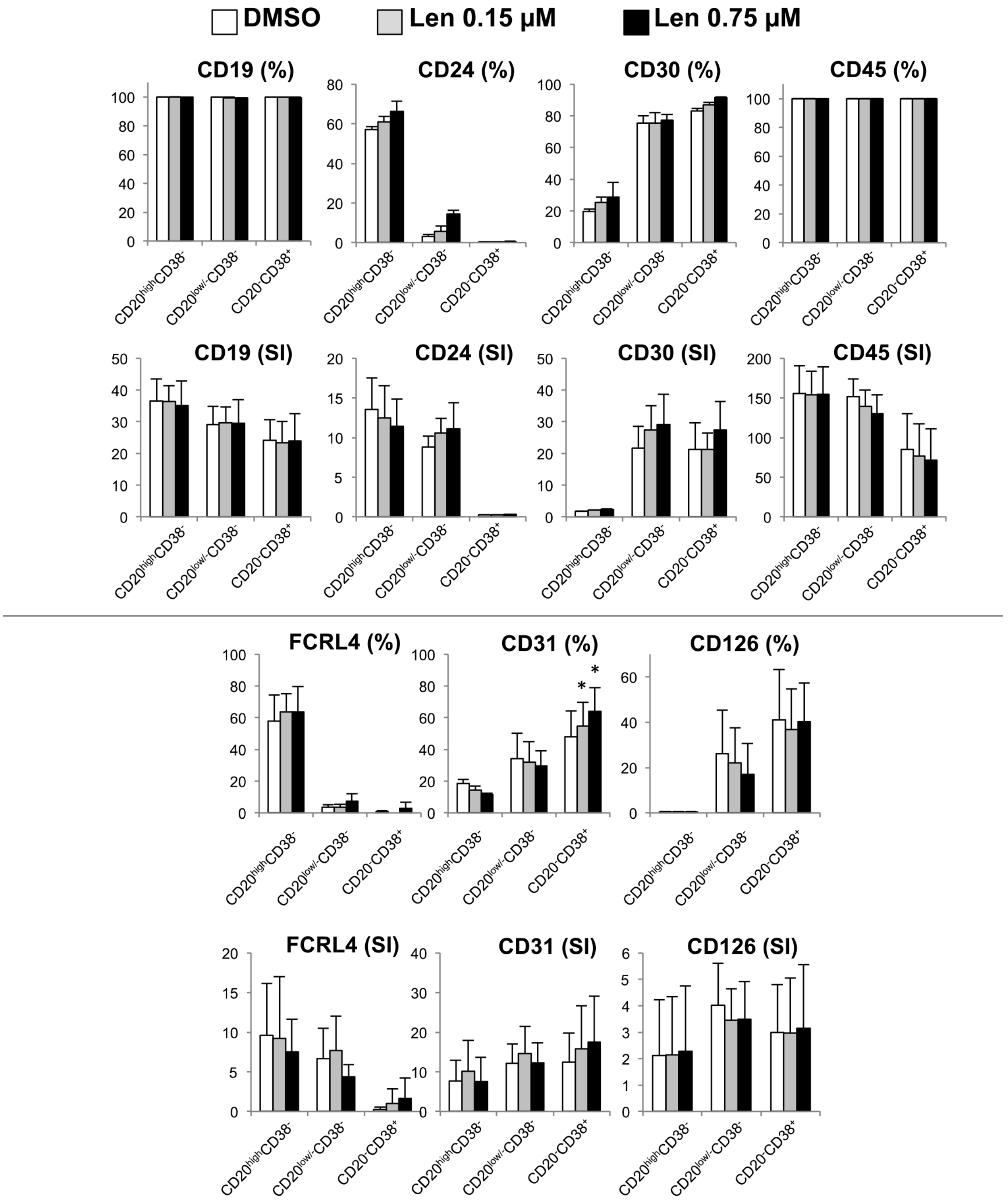

Supplementary Figure S4: Lenalidomide does not affect the membrane markers of B cells, preplasmablasts or plasmablasts. MBCs were activated with ODN and CD40L and cultured for 4 days with IL-2, IL-10, IL-15, and in the presence of graded concentration of lenalidomide or the largest DMSO concentration used to dilute lenalidomide (DMSO) as a control. Membrane markers were determined by FACS analysis at day 4. Results are the mean percentages and staining indexes (SI) ± SD determined on three separate experiments.

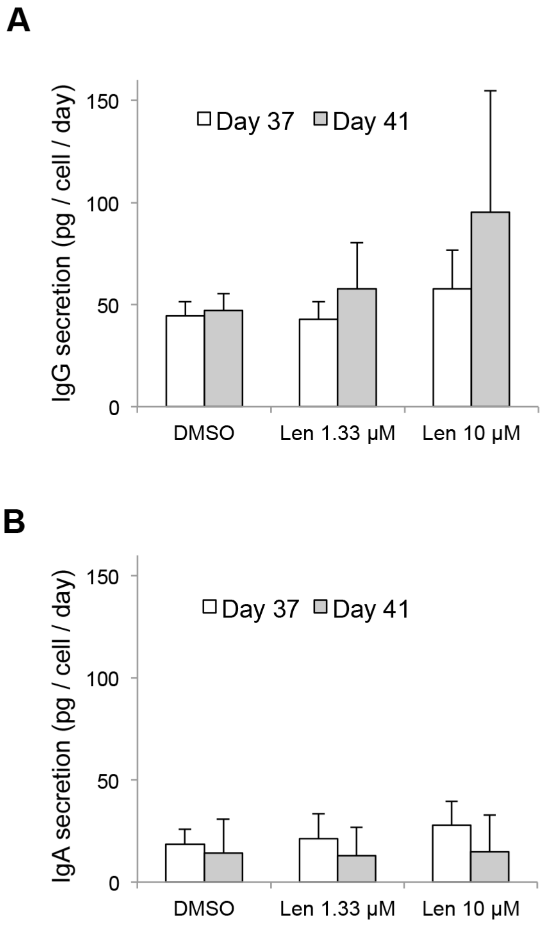

Supplementary Figure S5: Lenalidomide does not affect immunoglobulin production by long-lived plasma cells. LLPCs were generated from purified early PCs with IL-6, APRIL and SC-CM within 30 days and then cultured with increasing lenalidomide concentrations or the largest DMSO concentration used to dilute lenalidomide for 4 or 11 days. Fresh lenalidomide, DMSO, culture medium, cytokines and SC-CM were added after one week, by replacing half of the culture medium. Culture supernatants were harvested at day 34 or 41 and **A.** IgG and **B.** IgA production assessed by ELISA. LLPCs do not produce IgM. Results are the mean ± SD of Ig production (pg per cell and per day) determined in three separate experiments.

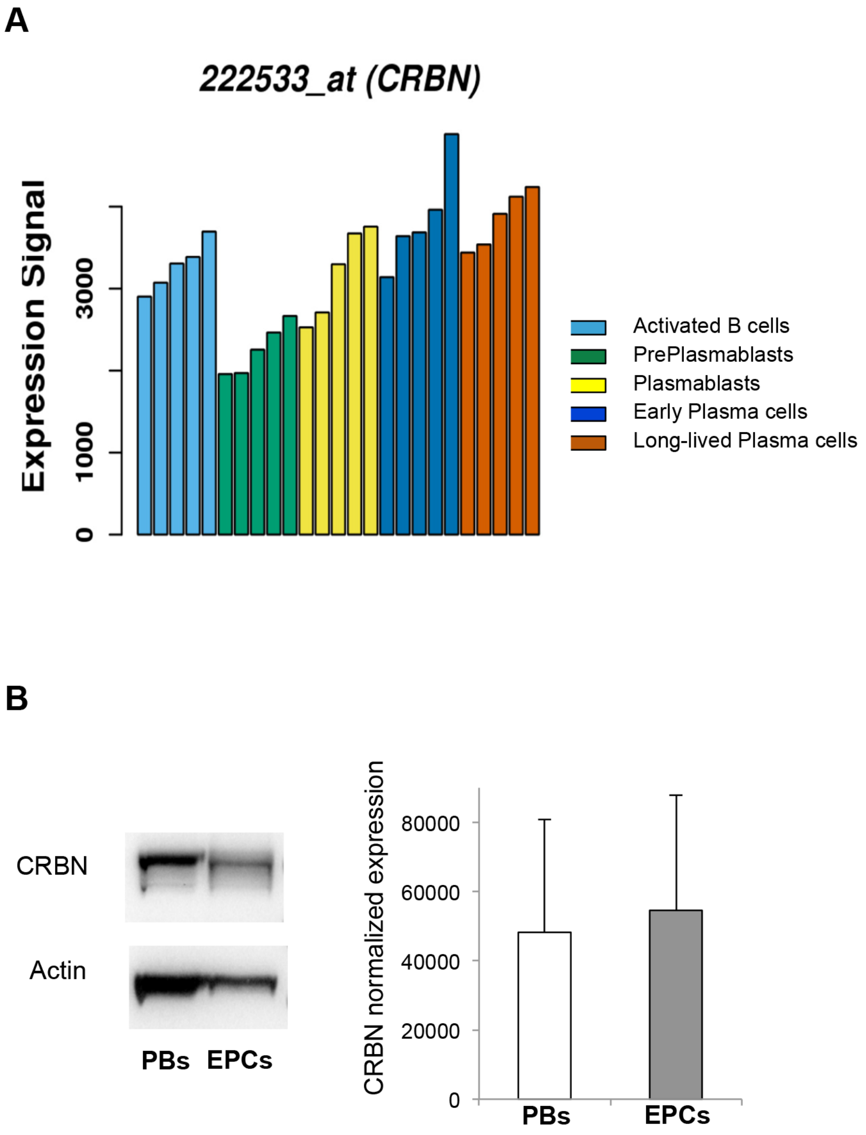

Supplementary Figure S6: CRBN gene and protein expression are comparable in the different cell types generated in the five-step plasma cell differentiation model. A. CRBN gene expression was determined using Affymetrix U133P microarrays as previously described [22]. Results are the MAS5 signal values in five samples for each cell group. **B.** CRBN protein expression was assessed in PBs and EPCs by western blot analysis. β actin expression was used as loading control. The blot is representative of two experiments and the histograms are the mean values of CRBN expression normalized to actin expression.

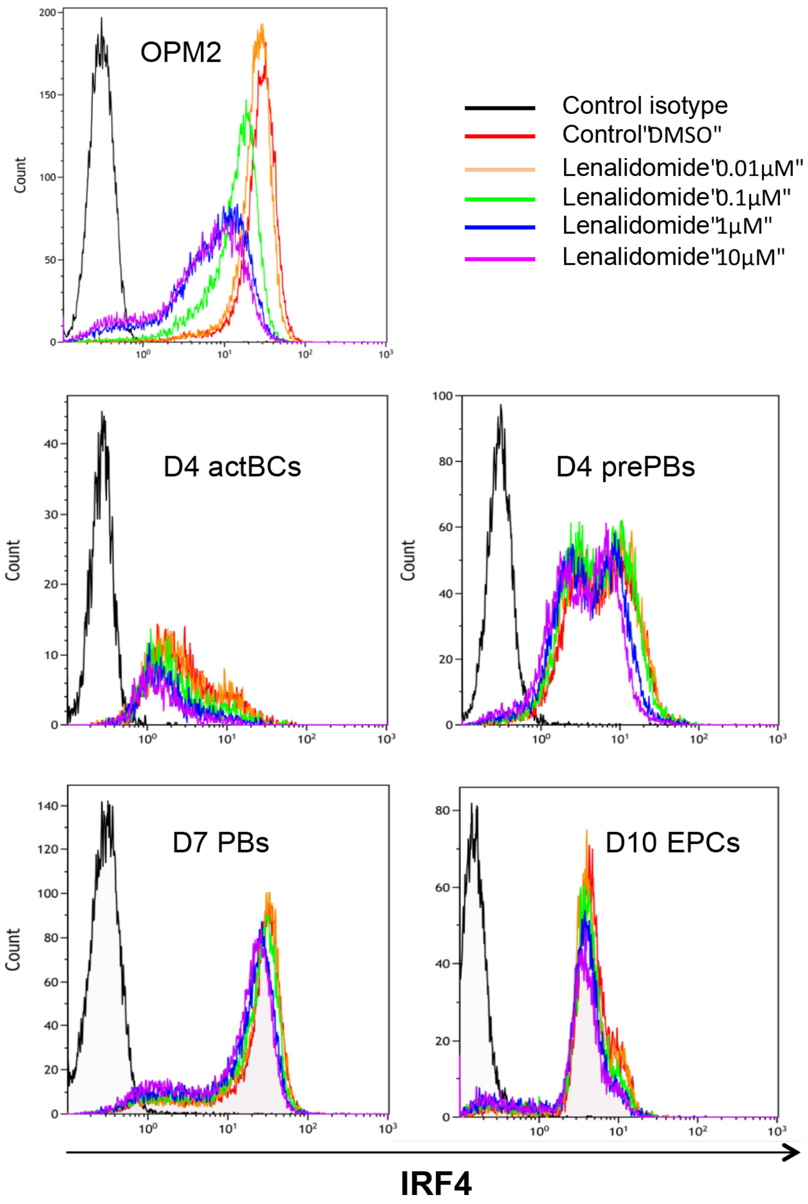

Supplementary Figure S7: Inhibition of IRF4 expression by lenalidomide. Cells were cultured in the presence of increasing concentrations of lenalidomide or the largest DMSO concentration used to dilute lenalidomide, as a control. Lenalidomide was added at the beginning of each step, as indicated in Supplementary Figure S1B. The human myeloma cell line OPM2 was cultured for three days. IRF4 expression in the different cell populations was detected by flow cytometry analysis at day 4, day 7 and day 10.
